# Digital tools to support the maintenance of physical activity in people with long-term conditions: A scoping review

**DOI:** 10.1177/20552076221089778

**Published:** 2022-04-11

**Authors:** Paul Clarkson, Aoife Stephenson, Chloe Grimmett, Katherine Cook, Carol Clark, Paul E Muckelt, Philip O’Gorman, Zoe Saynor, Jo Adams, Maria Stokes, Suzanne McDonough

**Affiliations:** 1 School of Health Sciences, 7423 University of Southampton, Southampton, UK; 2 130346 National Institute for Health Research Applied Research Collaboration Wessex, Southampton, UK; 3 Centre for Sport, Exercise and Osteoarthritis Research Versus Arthritis, Southampton, UK; 4 School of Physiotherapy, 402110 RCSI University of Medicine and Health Sciences, Dublin, Ireland; 5 National Institute for Health Research, 574429 Southampton Biomedical Research Unit, Southampton, UK; 6 Faculty of Health and Wellbeing, School of Health and Care Professions, 8629 University of Winchester, Winchester, UK; 7 Department of Rehabilitation and Sport Sciences, Faculty of Health and Social Sciences, 170790 Bournemouth University, Bournemouth, UK; 8 Physical Activity, Health and Rehabilitation Thematic Research Group, Faculty of Science and Health, School of Sport, Health and Exercise Science, 6697 University of Portsmouth, Portsmouth, UK

**Keywords:** Physical activity maintenance, behaviour change, Internet, chronic, digital health, multimorbidity

## Abstract

**Objective:**

This scoping review aimed to bring together and identify digital tools that support people with one or more long-term conditions to maintain physical activity and describe their components and theoretical underpinnings.

**Methods:**

Searches were conducted in Cumulative Index to Nursing and Allied Health Literature, Medline, EMBASE, IEEE Xplore, PsycINFO, Scopus, Google Scholar and clinical trial databases, for studies published between 2009 and 2019, across a range of long-term conditions. Screening and data extraction was undertaken by two independent reviewers and the Preferred Reporting Items for Scoping Reviews guidelines informed the review's conduct and reporting.

**Results:**

A total of 38 results were identified from 34 studies, with the majority randomised controlled trials or protocols, with cardiovascular disease, type 2 diabetes mellitus and obesity the most common long-term conditions. Comorbidities were reported in >50% of studies but did not clearly inform intervention development. Most digital tools were web-browser-based ± wearables/trackers, telerehabilitation tools or gaming devices/components. Mobile device applications and combination short message service/activity trackers/wearables were also identified. Most interventions were supported by a facilitator, often for goal setting/feedback and/or monitoring. Physical activity maintenance outcomes were mostly reported at 9 months or 3 months post-intervention, while theoretical underpinnings were commonly social cognitive theory, the transtheoretical model and the theory of planned behaviour.

**Conclusions:**

This review mapped the literature on a wide range of digital tools and long-term conditions. It identified the increasing use of digital tools, in combination with human support, to help people with long-term conditions, to maintain physical activity, commonly for under a year post-intervention. Clear gaps were the lack of digital tools for multimorbid long-term conditions, longer-term follow-ups, understanding participant's experiences and informs future questions around effectiveness.

## Introduction

Physical activity (PA) is an important part of maintaining both physical and mental health for people with one or more long-term conditions (LTCs).^1,2^ A LTC is a broad term for a range of physical and mental health conditions ‘that cannot at present be cured but can be controlled with medication or therapies’^3^ and is considered to last for more than 1 year.^4^ The World Health Organization (WHO) reports that ≥ 1.4 billion people worldwide are not active enough and an overall lack of progress at improving PA and reducing sedentary levels over the last 20 years.^4^ PA data from 2019/2020 for England highlights that 66.4% of adults were physically active to some extent each week.^5^ However, when compared with data from Sports England over the same period, 72.5%–75% of people with a disability or LTCs were inactive, defined as no activity in the last 28 days at two data points (May/November).^6^ Similar disparities in activity level between the general population and those with LTCs have also previously been reported in the research literature.^7,8^ Previous systematic reviews and guidelines have reported the benefits of PA for people with LTCs: to reduce some symptoms, prevent complications and maintain function.^7,9–11^

Digital tools as defined by WHO classifications, which includes digital and mobile technologies, such as websites, mobile device applications, telehealth and wearable devices^12^ and this is how the term will be used in this review. Digital tools offer great potential to support increasing PA and a wide range of previous systematic reviews have reported effectiveness at increasing PA levels in the short term for people with LTCs.^13–15^ The use of digital tools for this purpose also fits with a wider long-term agenda for digital tools to support existing services in the National Health Service (NHS) and more widely and has been found to be cost effective for some services.^16–18^ Digital tools may also be preferable to engaging with traditional services for some, given the flexibility of accessing support at a time that suits them, reducing transport-related issues^19^ and, since the COVID-19 pandemic, infection risk.^20^

Preliminary searches of the literature have identified few existing systematic reviews that focus on supporting people with LTCs to maintain PA using digital tools. Of those that do exist, their scope in terms of LTCs and multimorbidity, range of digital tools and maintenance outcomes is limited. For example, five systematic reviews, mostly with single condition cohorts (e.g. cancer survivors, obesity, chronic obstructive pulmonary disease (COPD), inflammatory arthritis and a mix of chronic conditions), found few studies reporting on the use of digital tools to support maintenance outcomes with either no or limited statistical evidence of effects.^21–25^ One reason for these findings may be that interventions that are designed to initiate change in behaviour, such as increasing PA, do not meet people's needs when attempting to maintain PA in the community for the long term.^26,27^

Maintenance of PA has been conceptualised by time and intensity of PA in different studies (regular activity or statistically significant change in behaviour over 1–12 months),^21,28,29^ behavioural automaticity or when the behaviour becomes the ‘dominant response’ in context.^30,31^ Time-based definitions for maintenance of PA have more recently focused on 3–6 months after the end of the intervention.^21,28,29^ Given the limited number of studies reporting maintenance of PA and the heterogeneity between studies in previous reviews,^21,24^ we concluded that a novel scoping review would be appropriate to explore the range and depth of available literature in this area,^32,33^ to direct future systematic reviews and/or primary research questions.

The use of theory in the development of behaviour change interventions, as part of a wider programme theory approach to intervention development^34^ is associated with increased effectiveness.^35^ Consequently, it is important to understand whether the theory has been used to develop digital tools, and if so, which theories are associated with the maintenance of PA. Identifying the theoretical basis and use of behaviour change techniques (BCTs) as intervention components will help support the replication of effective strategies and provide evidence to inform future intervention development.^36^ Key theories that have previously been associated with the maintenance of health behaviours are theories of self-regulation,^28,37^ and self-determination theory.^38^

Furthermore, the increasing focus on digital health in healthcare systems, both before the COVID-19 pandemic and especially since,^16,39^ has meant that clinicians and commissioners need to understand what evidence-based digital tools are available for implementation. This scoping review will systematically map the research undertaken and planned in this area to identify tools that may be suitable for replication and to identify any existing gaps in knowledge.

This review aimed to answer the following objectives:

1. What is the ‘extent (size), range (variety) and nature (characteristics) of the evidence’^40^ on digital tools to support the maintenance of PA for people with one or more LTCs?
2. What theoretical underpinnings are used in digital tools to promote the maintenance of PA?

## Methods

This review was conducted in accordance with guidance from the Preferred Reporting Items for Scoping Reviews (PRISMA-ScR),^40^ the Joanna Briggs Institute,^41^ and existing scoping review frameworks.^33,42^ The protocol for this review is available from Protocols.io.^43^

### Eligibility criteria

The eligibility criteria for LTCs, PA and digital tools are shown in Table 1. The list of included LTCs was based on The Quality and Outcomes Framework (2017/2018)^44^ and the National Institute for Health and Care Excellence PA pathways.^45^ Broader terms (e.g. ‘chronic’, ‘long-term condition’, ‘multimorbidity’) in searches and studies were included if one or more of the LTCs were reported (Table 1). A small-scale pilot identified that some studies included defined LTCs as a subset of a larger sample. An a priori decision was taken to include these studies where all the other eligibility criteria were met, and results were charted for the relevant LTCs if possible. Cancer and low back pain were excluded due to existing recent reviews.^21,46,47^

**Table 1.** Eligibility criteria for study inclusion.

**Dates**	**2009–2019 for full-text studies**
	**2017–2019 for abstracts (to avoid duplication with full texts)**
**Long-term conditions included**	Asthma
	Cardiovascular disease including atrial fibrillation, hypertension, heart failure, peripheral arterial disease, secondary prevention^a^ of coronary heart disease
	Chronic kidney disease
	Chronic obstructive pulmonary disease
	Dementia
	Depression
	Type 1 or 2 diabetes mellitus
	Epilepsy
	Mental health
	Myocardial infarction: secondary prevention^a^
	Obesity
	Osteoarthritis
	Osteoporosis
	Rheumatoid arthritis
	Stroke/transient ischaemic attack
**Long-term conditions excluded**	Cancer, low back pain
**Physical activity inclusion**	Adults not meeting ≥ 150 min MVPA per week
**Outcome timing**	Must have measured a physical activity outcome at least 3 months post the end of the intervention
**Physical activity exclusion**	Studies that report a reduction in sedentary time only
**Digital tools included** ^12^	Targeted client communication such as email or other messaging interventions. Web-based intervention
	Untargeted client communication, such as web-based or software-based interventions, including video
	Client -to-client communication, such as digital peer support group
** **	Personal health tracking, such as smartwatches or other activity trackers with a visual display
	Telemedicine systems with visual display for user
	On-demand information services to clients such as digital sources of information
	Client financial transactions such as digital incentive management
	Other tools that included exergaming, gamification
**Digital tool excluded**	Pedometers/accelerometers used alone without connection to another digital tool

^a^ Preventing progression of an established condition.

MVPA: Moderate-to-vigorous physical activity.

Studies with adults (≥ 18 years) who were not currently achieving the recommended levels of PA, based on the United Kingdom (UK) PA guidelines (≥ 150 min of moderate-to-vigorous activity (MVPA) per week)^48,49^ were included. Maintenance was defined as at least 3 months after the end of the intervention. While attempts were made to include studies with no contact during the maintenance period, it was recognised that this would have been too restrictive. Instead, studies were included when there was either no contact with the intervention or where a lesser version of the intervention was employed during the maintenance period. This information was charted in accordance with guidance.^33^

Digital tools were defined using the classification of digital health interventions from the WHO^12^ (Table 1). All study designs were eligible for inclusion including quantitative, qualitative and mixed methods studies, protocols and conference abstracts.

### Information sources

Preliminary searches were conducted in Cumulative Index to Nursing and Allied Health Literature (CINAHL) and Medline to establish appropriate search terms. The search strategy (Supplemental material 1 Appendix A) was developed alongside an academic librarian, members of the research team and based on previously published search terms.^21,28,50^ The search strategy was made up of keywords (e.g. digital, physical activity, maintenance and the list of LTCs (Table 1)) as well as synonyms of these terms, which were connected using Boolean operators. This search strategy was initially set up to support a search of the Medline database, before being adapted to accommodate the syntax of other databases. Comprehensive searches were undertaken in CINAHL, Medline, OVID EMBASE, IEEE Xplore, PsycINFO, Scopus and Google Scholar (to capture grey literature). Clinical trial registries (e.g. International Prospective Register of Systematic Reviews, International Standard Randomised Controlled Trial Number database, International Clinical Trials Registry Platform, European Union clinical trials register, and Clinicaltrials.gov) were also searched to ensure that ongoing and recently completed studies were not missed. Databases were searched from 2009 to 2019, to follow on from a previous review that searched up to 2009, finding only one digital tool.^29^ Searches were conducted between 17 and 28 January 2020.

### Study selection

**Figure 1.**
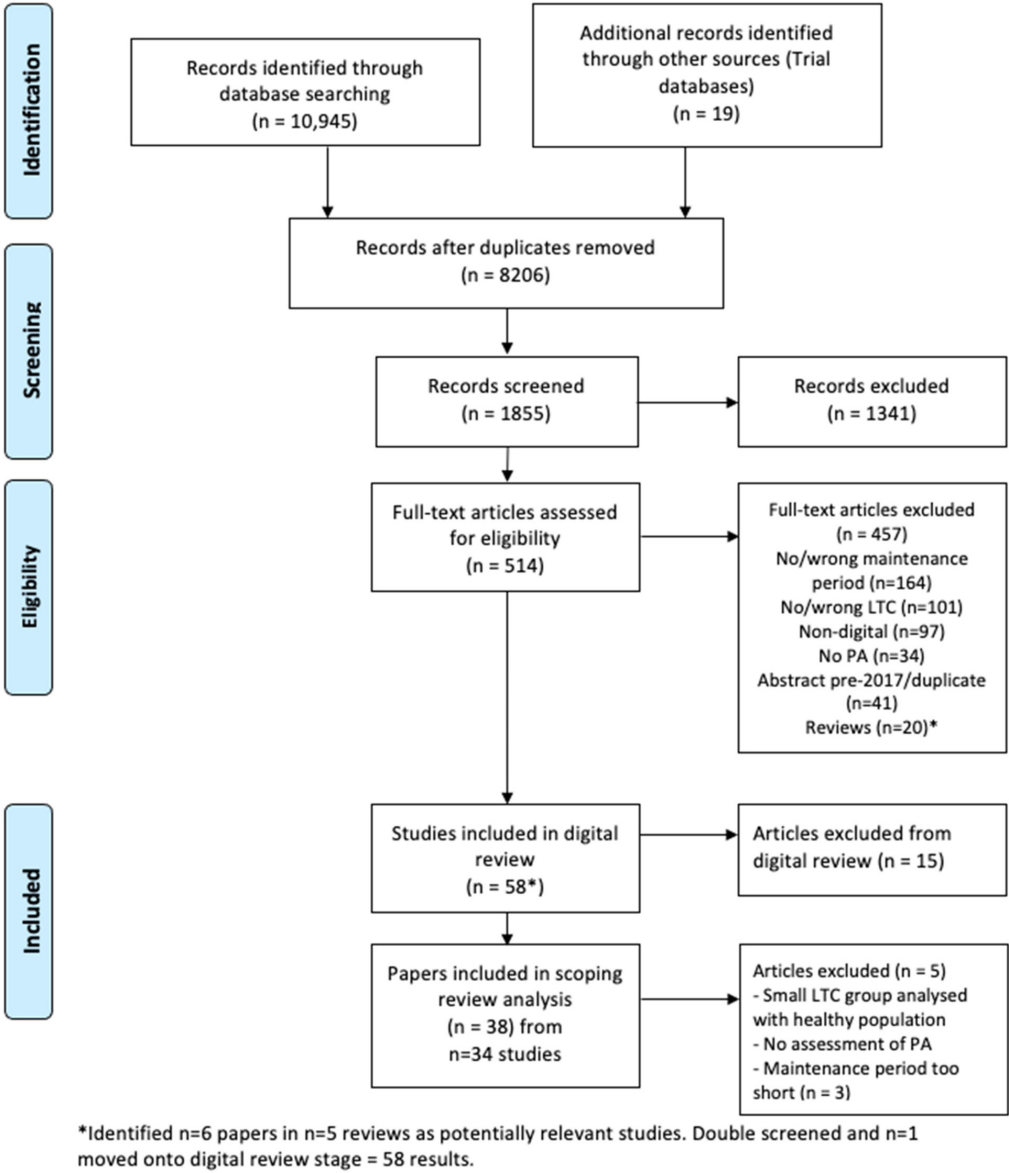
PRISMA flow diagram^35^ for review phases, including results identified, excluded and reasons for exclusion.

Search results were transferred into endnote (Clarivate Analytics, Boston, MA). Five percent of titles were initially screened independently by two reviewers (PC, SMcD) and then discussed to determine agreement, before the remaining titles were screened by PC. A random 5% sample of titles and abstracts were screened initially by PC and SMcD, with clarifications made to the eligibility criteria. Results were transferred into the Covidence software (Veritas Health Innovation, Melbourne, Australia) for title/abstract screening by the team. Each reference was screened by four groups of two independent reviewers (PC, CC, SMcD, PM, CG, AS, JA, ZS), with conflicts highlighted through the software and decided by a verifier. The research team (PC, PM, KC, CC, CG, AS, POG, ZS) undertook the same process for full-text review but were required to select a reason for exclusion from the predefined eligibility criteria listed in Covidence. The final process of screening was undertaken by four members of the team working in two pairs (PC, SMcD; POG, AS) to determine whether the interventions were predominantly digital, based on criteria established through consensus for a related systematic review involving AS, SMcD (online Supplemental material Appendix B). Results were screened and discussed to confirm eligibility. Literature reviews that were identified as relevant to the eligibility criteria in the search results had their included studies checked against the list of included and excluded studies from the scoping review. Studies that were found to be eligible for inclusion and had not already been identified through database searches were screened and the literature reviews were excluded (Figure 1).

### Data charting process and data items

Data from the included studies were charted into an Excel sheet developed a priori based on guidelines^38^ and previous studies.^21,51^ This included study characteristics such as design, location, setting, primary LTC (Table 1) and comorbidities of any kind, in addition to number, age and gender of participants. Intervention description and length (defined in Table 1), inclusion/exclusion, maintenance period (≥ 3 months after the end of the intervention) and any reported access to elements of the intervention during this period were recorded. Type and tool used to measure PA (objective or self-reported), and theoretical underpinning (behaviour change theory or BCTs explicitly mentioned, as the BCT taxonomy^36^ was not used for extraction purposes due to resource limitations) were reported (full list in Supplemental material 1 Appendix C).

The charting form was piloted using one of the included papers before data extraction began, to clarify understanding of the categories. Eight members of the team were divided into pairs to undertake data extraction (PC/KC, SMcD/ZS, CC/PM, POG/AS) with the included studies divided between them. Each reviewer independently read and extracted data into the charting form, before meeting with the other reviewer to discuss and agree on the final extraction. Where appropriate, reviewers contacted study authors to clarify additional detail. In accordance with scoping review guidelines, critical appraisal was not undertaken.^32,40^

### Synthesis

The charting forms were collated into one excel sheet by PC, before collation and summarising of the data based on the objectives by PC, POG, AS, SMcD. The charted data were reviewed, summarised and clarified with the original sources. These summaries were discussed to identify the most appropriate way of presenting the results, before being sent to the wider team for review and presented at a team meeting. Data were presented descriptively using frequencies and measures of central tendency. Characteristics of the interventions included description, hardware used, intervention components, including non-digital components, type of digital tool,^12^ (Table 1) and length of intervention. The longest length of maintenance period and any access during to the intervention during this period were synthesised. Reports of theoretical underpinnings of the interventions were collated. Theories were only extracted if they were listed as one of the 83 theories of behaviour change from the ABC of behaviour change theories,^52^ developed by an expert group to be relevant to the design of interventions. BCTs were collated from studies that reported use of the BCT taxonomy^36^ or its precursor by Abraham and Michie.^53^

## Results

Database searches identified 8206 results (Figure 1). Title review resulted in the exclusion of 6351 results. The team reviewed 1855 titles and abstracts, which resulted in 514 potentially relevant studies for full-text review. Reasons for exclusion at this stage included a lack of maintenance period, measurement of sedentary time only and studies where a pedometer was the only digital tool. During the full-text review, 457 citations were excluded, predominantly for not meeting the maintenance definition (n = 164), not including the defined LTCs (n = 101), or not including a digital tool (n = 97). PA outcomes were not included in 34 results, while 41 citations were abstracts from before 2017 and therefore excluded. The team identified six potentially relevant citations from five reviews. In total, 20 reviews were excluded at this stage. After screening the six citations from the reviews, one was moved onto the digital review stage. Fifteen further citations were excluded during the digital review stage. A further 5 citations were excluded during the data extraction stage, leaving 38 results, from 34 studies to be included in the review (Supplemental material 1).

### Study characteristics

Of the 38 included papers, 19 were either randomised controlled trials (RCTs)^54–71^ or used a quasi-experimental design,^72^ 14 were protocols for RCTs,^73–86^ three were pilot or feasibility studies,^87–89^ one study used a correlational design^90^ with a single group, and one was a mixed-methods process evaluation^91^ linked to one of the protocols^73^ and RCTs.^62^ Studies were mostly undertaken in Europe, with The Netherlands hosting the most studies (8/34), although the largest recruited sample sizes were reported in studies from North America and Australia.^56,57,61,70^ Sample sizes at baseline ranged from n = 20–2000 overall (including anticipated samples from protocols). More than 60% of studies were undertaken since 2016 indicating increasing interest in this area. This is further exemplified by the identified protocols, which target the recruitment of a greater number of people with LTCs for future trials (Supplemental material 1).

**Figure 2.**
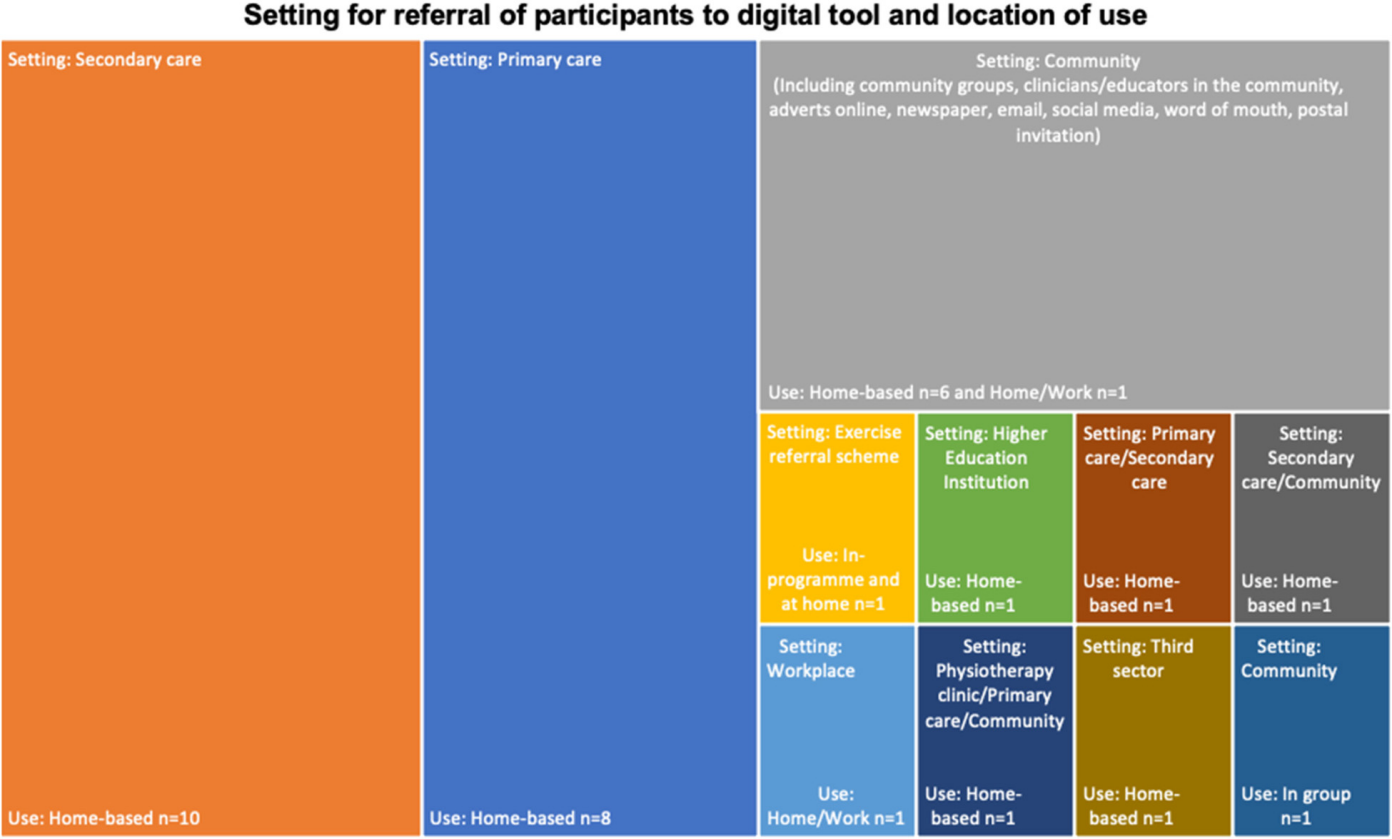
Setting for referral of participants to digital tool and location of use. (Figure 2 shows 33 studies rather than 34 due to the setting being unclear in Barnason et al.^90^)

Cardiovascular disease was the most common LTC across the sample of studies (10/35), followed by type-2 diabetes mellitus (T2DM) (7/35) (Table 2). A greater number of females were recruited overall, although three of the RCTs reported that females made up a much smaller proportion of the overall sample (10% to 20%). Mean age of participants ranged from 33.9 to 66.3 years in the intervention groups. The most common setting for referral or recruitment to use the digital tool was secondary care (10/33), followed by primary care (8/33) (Figure 2). Seven studies were defined as being undertaken in the community setting, which included community groups, referral from community-based clinicians, as well as adverts, postal invitations and word of mouth. Other settings for the included studies are shown in Figure 2. Most interventions (29/33, 88%) were designed to be used at home, while some were designed for use in a work setting (n = 2) or within a community/local authority programme or group (n = 2).

**Figure 3.**
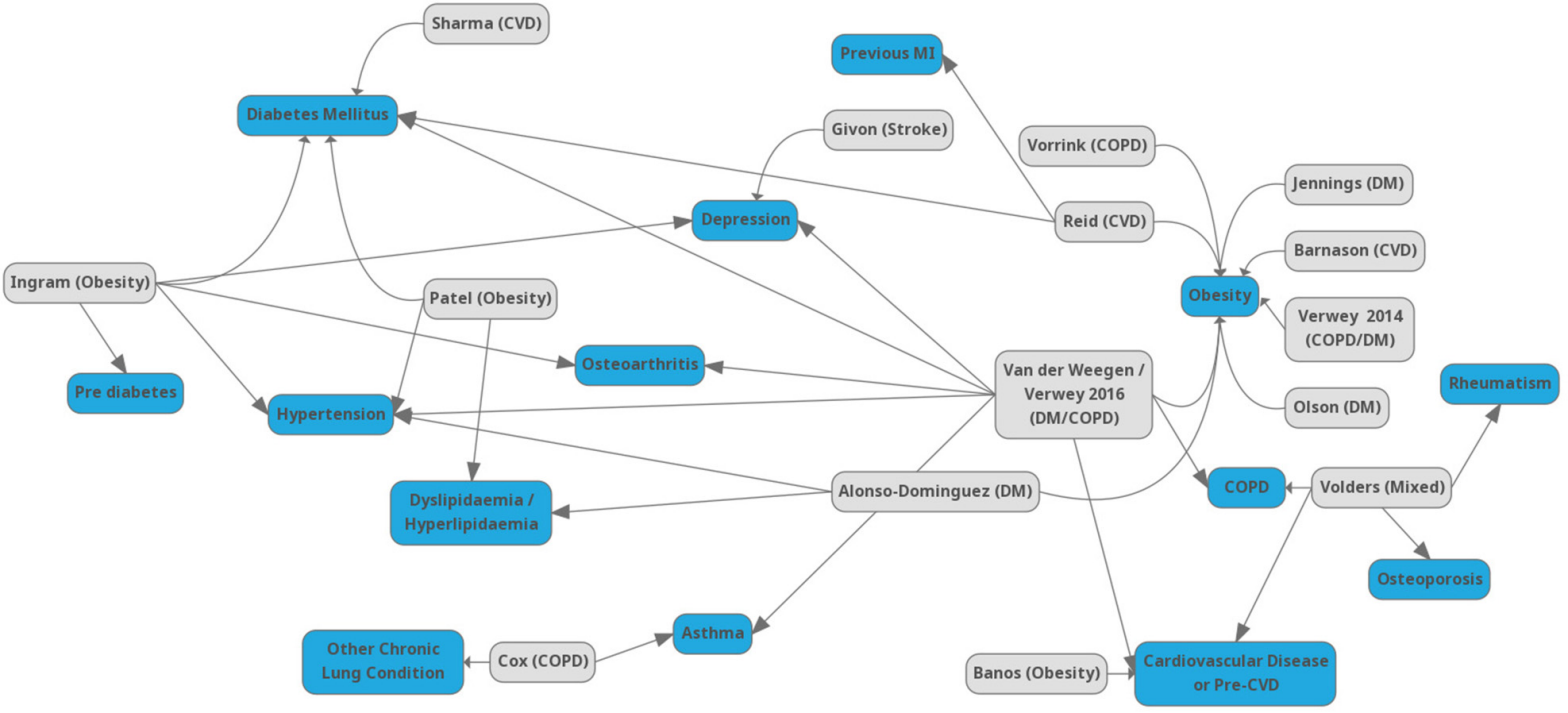
List of studies with primary long-term conditions (LTCs) and linked comorbidities.

Types of comorbidity were reported in 16 studies, while a further three studies reported comorbidities in their samples, but did not define the number or condition/s. Figure 3 shows the studies and primary LTC, with linked comorbid condition/s. The most commonly reported comorbidities were obesity and T2DM. There was little indication in the majority of studies that the interventions were amended in any way to support these comorbidities. Two studies^82,85^ may have adapted the intervention to account for the comorbidities reported, although this was not stated clearly.

Grey boxes show the first author of the included study and primary LTC in brackets. Blue boxes show comorbidities. Bossen et al.^58^ is not shown as did not report specific comorbidities.

### Characteristics of the digital tools

Full details of the interventions are shown in Supplemental material 1. Digital tools were predominantly web-browser based (13/34) or used the web alongside a wearable/activity tracker or pedometer (5/34). Telerehabilitation interventions were used in a further five studies, while a gaming device or an intervention that used gaming elements was used in four studies. Mobile device applications (apps) were used in three studies. Short message service (SMS) interventions with and without an activity tracker were used in two studies, and wearable devices with a connection to a website or app were included in two studies. There was a wide range of intervention lengths from 2 weeks to 12 months.

All interventions were delivered digitally, although most (22/34) included a healthcare professional (HCP) or other facilitators as an active part of the wider intervention (Table 3). A further three studies^57,59,65^ included an HCP or facilitator to introduce the digital tool to participants and/or set goals and provide feedback. One study^62^ was app-based linked to a website that allowed HCPs to set goals and monitor progress. Eight interventions did not include any active contact with an HCP or facilitator.^58,61,63,72,79,85,86,88^ The most common intervention components, reported by the author's intervention descriptions, were the use of motivational messages delivered either digitally, over the telephone or in person (21/34) and goal setting (18/34).

**Table 2.** Study characteristics.

**Study location (n = 34)**	Europe	17 (50.0%)
	**North America**	**8 (23.5%)**
	**Asia**	**4 (11.8%)**
	**Australia**	**4 (11.8%)**
	**Mixed continent (Europe/Asia)**	**1 (2.9%)**
**Publication date (n = 38)**	**2009–2012**	**4 (10.5%)**
	**2013–2015**	**11 (29.0%)**
	**2016–2019**	**23 (60.5%)**
**Primary LTC (n = 35)**	**Cardiovascular disease (CVD) (including hypertension, heart failure, ischaemic heart disease, angina, coronary artery disease)**	**10 (28.6%)**
	**Type 2 diabetes mellitus (T2DM)**	**7 (20.0%)**
	**Obesity**	**6 (17.1%)**
	**Chronic obstructive pulmonary disease**	**2 (5.7%)**
	**Stroke**	**2 (5.7%)**
	**Osteoarthritis**	**2 (5.7%)**
	**Depression**	**1 (2.9%)**
	**Rheumatoid arthritis**	**1 (2.9%)**
	**No single LTC reported**	**1 (2.9%)**
	**Mixed (n = 1 COPD, rheumatism, osteoporosis, chronic heart disease, musculoskeletal; n = 2 T2DM, COPD)**	**3 (8.5%)**

Primary LTC included n = 35 papers due to the protocol of one RCT^73^ reporting one LTC (COPD) and the subsequent RCT and process evaluation^62,91^ reporting a mix of conditions (type-2 diabetes mellitus and COPD).

COPD: chronic obstructive pulmonary disease; LTC: long-term condition; RCT: randomised controlled trial.

**Table 3.** Healthcare professional/facilitator involvement in the study interventions.

	Active part of intervention^a^	Monitoring	Referral to tool or set up goals/feedback	No active intervention contact^b^
**Vorrink, 2016**		•	•	
**Thorup, 2016**			•	
**Lorig, 2010**	•			
**Jones, 2016**	•			
**Hurkmans, 2010**	•			
**Lari, 2018**				•
**Jaarsma, 2014**	•			
**Jennings, 2014**				•
**Hawkins, 2019**	•			
**Dor-Haim, 2019**	•			
**Devi, 2014**	•			
**Barnason, 2016**	•			
**Harrison/Patel, 2019**	•			
**Bouwers, 2017**	•			
**Bossen, 2013**				•
**Bonn, 2018**	•			
**Barry, 2011**	•			
**Fife-Schaw, 2014**	•			
**Avila, 2019**	•			
**Olson, 2015**				•
**Alonso-Dominguez, 2017/2019**	•			
**Verwey, 2014/2016** **Van der Weegen, 2015**	•			
**Kloek, 2014**	•			
**Ingram, 2018**	•			
**Strom, 2013**			•	
**Vorderstrasse, 2017**				•
**Reid, 2012**			•	
**Volders, 2019**				•
**Lubans, 2009**	•			
**Givon, 2016**	•			
**Yang, 2017**	•			
**Sharma, 2019**				•
**Cox, 2018**	•			
**Banos, 2015**				•

^a^ Active direct involvement during intervention period.

^b^ May include automated reminder messages delivered digitally.

### Maintenance period and measurement of PA

**Figure 4.**
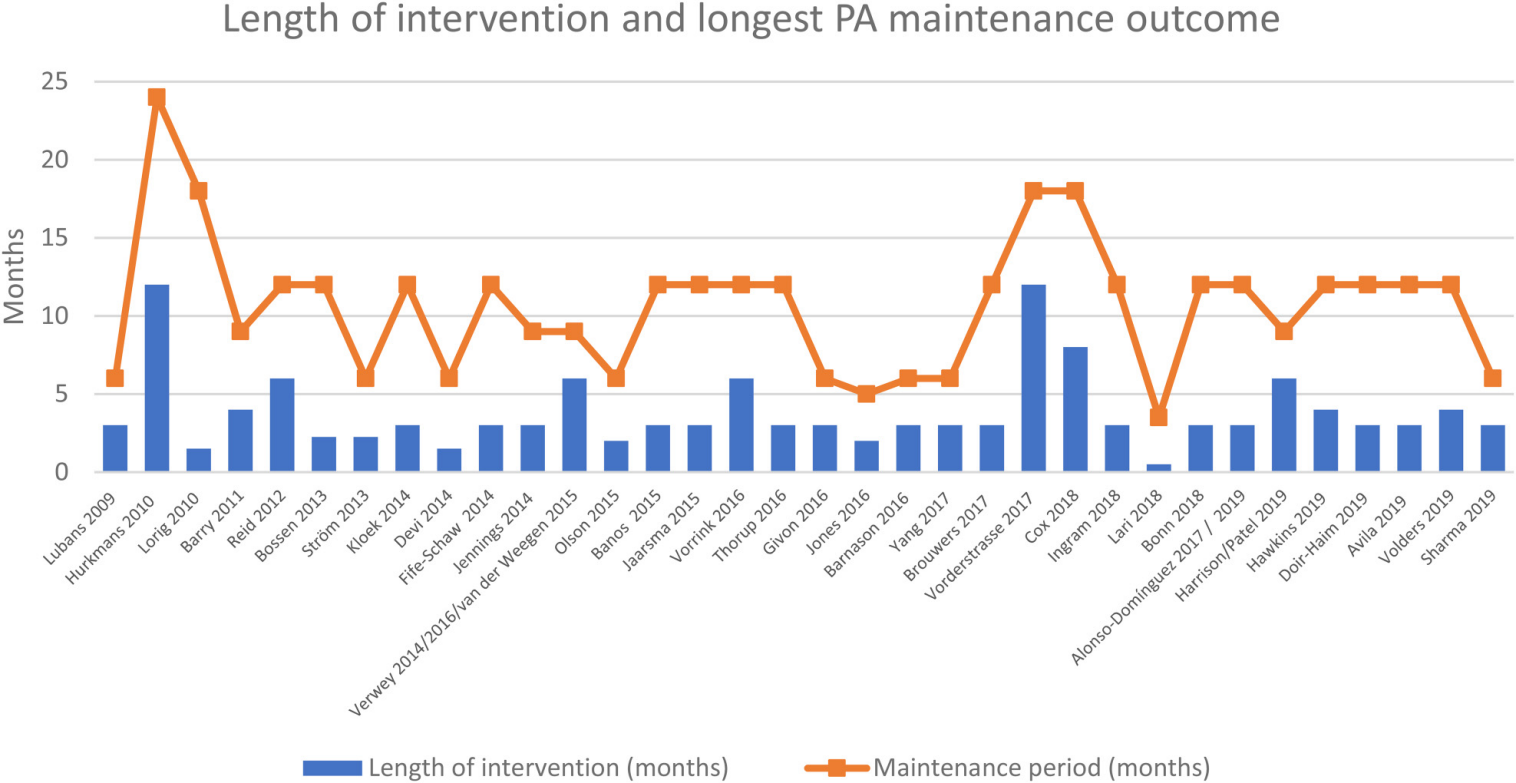
Length of interventions and related longest physical activity (PA) maintenance outcomes.

Maintenance periods ranged from three to 12 months post-intervention with 9 months (11/34) and 3 months (9/34) the most commonly reported. Figure 4 shows the point at which the longest maintenance outcome was recorded for each study, in relation to the length of the intervention. Most studies reported no access to the intervention during the maintenance period (18/34) or access to a lesser version of the intervention (10/34). Six studies were unclear. PA was most often objectively measured (19/34) alone or alongside a participant-reported outcome measure (PROM) (8/34). A further seven used a PROM alone. The most commonly used devices for measuring objective PA were the Actigraph accelerometer (10/27), SenseWear Armband (4/27), FitBit step counter (3/27) and GENEActiv accelerometer (2/27). The most commonly used PROMs were the International Physical Activity Questionnaire (IPAQ) (5/15) and the Short Questionnaire to Assess physical activity (SQUASH) (3/15). (Other devices or PROMs used are shown in Supplemental material 1).

### Theoretical underpinnings of interventions

Interventions were predominantly delivered using digital tools, however, some also included non-digital components (Supplemental material 1, Table 3). Theoretical underpinnings are presented for the whole study intervention as it was not possible to isolate digital/non-digital components. Fifteen interventions reported in 18 papers clearly articulated the use of behaviour change theory in the development of the intervention. Two of these interventions also had other theories associated with them, but it was unclear whether these were used in the intervention development process. A further seven studies reported the use of theory, but it was unclear whether this was specifically related to the development of the intervention. In the remaining 12 studies, there was either no theoretical underpinning reported or limited evidence to suggest the use of theory. The most commonly cited theories were social cognitive theory (SCT) (n = 5, + 1 unclear use as an underpinning), the transtheoretical model (TTM) and the theory of planned (TPB) behaviour (both n = 3, + 1 unclear use as an underpinning). BCTs^36^ were mentioned in four studies, but it was unclear whether they were specifically used in the development of the intervention.

## Discussion

To our knowledge, this is the first scoping review to map the range and breadth of digital tools to support people with a wide range of the most prevalent LTCs to maintain PA. Over the last 20 years, our review shows that web-based digital tools continue to predominate with more recent emergence of gamification, apps and virtual environments. Interventions continue to be aimed at supporting people with a single LTC, even though a large proportion of participants also had comorbidities. Most participants were from younger age groups. The use and description of theory in the development of the tools were limited, with a lack of transparent reporting, and most studies highlighted the need for human engagement to support their use.

A novel finding of our review compared to previous reviews is the wealth of evidence we identified. There is a significant body of evidence (n = 34 studies), demonstrating the benefit of conducting a scoping review across multiple LTCs. Previous reviews with a focus on single LTCs have reported minimal use of digital tools to support the maintenance of PA,^21–23^ while others that have focused on digital technologies for LTCs report minimal or no use of outcomes in the maintenance period,^13,24,25^ including for one of the excluded conditions, low back pain.^47^ Our results demonstrate an increasing interest in the use of digital tools to support people with LTCs to maintain PA over the review period and particularly since 2016. This may reflect the increased interest and guidelines advocating digital health strategies within Europe over the same period.^16,92,93^ Most of the identified digital tools used the Internet in some form, either as the primary delivery modality, e.g. web browser-based interventions; or in an accessory capacity, such as providing visual PA metrics through an activity monitor or app. The present review identified only three studies that developed apps, which is surprising given the exponential increase in the number of available apps from commercial app stores, although many are not designed specifically for people with LTCs.^94^ However, some apps are reported to have a limited evidence base^95^ and it is therefore likely that our review would not have captured them, as development work is unlikely to have been published in academic journals.

The use of theory was effectively described in fewer than half of the identified studies, and identifying it proved to be a difficult task, due to inconsistencies in reporting, and we were unable to separate the digital and non-digital theoretical components. SCT, TTM and TPB were the most commonly reported theories, which is similar to other reviews of PA maintenance interventions.^21,22,25^ Guidance on intervention development suggests that a theoretical underpinning is best practice and is associated with greater effectiveness,^37,96,97^ while other studies show equivocal outcomes across the age and condition spectrum.^98,99^ Michie and colleagues developed a BCT taxonomy to support fidelity in the delivery of an intervention and to identify the effective components for behaviour change to improve future intervention development.^36^ BCTs were only reported in four studies, although their specific use as an ‘active ingredient’^36^ was less well described. Inconsistent description of intervention components has previously been reported,^100^ including in eHealth interventions for people with cardiovascular disease (CVD),^101^ and was not described in a review of web-based interventions for low back pain,^47^ reducing the potential for replication and translation of findings.^102,103^ Given these identified limitations, we intend to explore the effective components of interventions in a future systematic review using intervention component analysis.^104^

While digital tools made up the primary component of interventions, additional human support (via HCP or other facilitators) was identified in most studies which have implications for staff resources needed to scale up potential solutions. Key aspects of digital interventions in our review, i.e. motivational messages and goal setting, often supported by HCPs or other facilitators, have been reported in previous reviews to support PA for people with and without LTCs.^21,24,95^ There is debate as to whether human support is needed. Some highlight the importance of human support to promote adoption and follow-up of Web 2.0 tools, defined as ‘participatory internet interventions’.^2, 25^ Others, including a review of web-based interventions for low back pain have reported mixed results in terms of additional support.^47^ Clearly, there are pros and cons to the involvement of HCPs alongside a digital tool: it can help to reduce anxiety and increase feelings of support^105^ and build self-efficacy,,^106^ or it may lead to a reliance on HCP input to self-manage LTCs.^107^ While digital tools and services are often considered to be part of the solution for reducing pressure on health services,^107^ these findings suggest that human input may still be required to support their use, further work is needed to better understand how to optimise digital tools through HCP support. Challenges remain, both in terms of ensuring the availability and digital capability of staff to meet the need of people when scaling up interventions.^108^

This scoping review identified digital tools which were designed for people with the most prevalent single LTCs. Previous systematic reviews in this area have also focused on the most prevalent conditions, including obesity, COPD, inflammatory arthritis and cancer survivors.^15–18^ Comorbidities were reported in more than half of the identified studies in the present review, but there was limited evidence that the digital tools had been developed to take account of the impact of these conditions. Comorbidities were rarely reported in studies included in previous reviews, but when they were reported, approximately half of the digital tools were designed to support these comorbidities.^21–24^ A future systematic review could explore whether this influenced the effectiveness of digital interventions.

In the present review, the upper age range of included participants was not representative of the largest proportion of people with LTCs, who are aged 65–99, based on the UK Office for National Statistics (ONS) data^109^ and Irish Health Survey data from 2019.^110^ This emphasises a key limitation of the current evidence and is particularly disappointing, given the greater age-associated prevalence of LTCs such as CVD and T2DM, and the greater mortality rates related to CVD and T2DM as multimorbidities.^111,112^ Furthermore, given projections of ageing populations in Europe over the next 30 years,^113^ it is increasingly important to include older adults in technology-related research to ensure that the needs of these groups are met. However, as is evident from the present findings, recruiting older adults is often difficult.^114^ Reasons for this are reported to include a lack of interest, transportation issues (when required) and advice from family or clinicians against participation.^114^ Others have highlighted that while older adults are open to the use of technologies, barriers to involvement include a lack of clear information and user support.^115^

This scoping review only included studies that reported a PA maintenance outcome, with the majority reporting outcomes at either 9 months or 3 months after completion of the intervention. The longest maintenance outcome was reported at 12 months, which aligns with some of the previous reviews in this area, both for digital and non-digital interventions.^23–25,29^ However, others have reported maintenance outcomes of between 3 and 5 years,^21,22^ although predominantly for non-digital interventions. Similar findings have been reported recently for non-digital interventions aiming at supporting longer-term PA^116^ Future digital studies should therefore focus on reporting longer-term outcomes to understand their effectiveness over these longer periods.

## Strengths and limitations

The use of a scoping review methodology has enabled the identification of a coherent body of evidence of both developed and planned interventions, and their components, which would not have been possible with a systematic review. Indeed, many of the existing systematic reviews focus on a single condition and/or a narrow interpretation of digital tools.^21,22,24^

A strength of this scoping review is also the wide lens used to map literature across 18 LTCs as the first step to inform a systematic review and/or design of future digital interventions for maintenance of PA in people with multimorbidities. This broad approach also extended to the inclusion of digital tools and the use of a conservative definition for maintenance,^21,29^ enabling a wide range of literature to be identified in this area. However, non-English language studies were not included, which may have meant that studies were missed, particularly given the number of studies identified in Europe. The maintenance definition was purposefully inclusive; however, studies were identified during the screening process that was highly relevant but did not exactly meet this definition and were subsequently excluded. Furthermore, our maintenance definition will have excluded interventions designed to be used during the maintenance period.

This review also aimed to explore the experiences, barriers and facilitators for people with LTCs to using digital tools to maintain PA. Unfortunately, we only identified qualitative data in one of the RCTs^89^ and were therefore unable to address these objectives. On reflection, it may have been prudent to develop a second search strategy that focused on identifying qualitative and process evaluations of interventions or to undertake a snowballing approach after identifying the studies included.

Given the pace of change in this area, it is likely that a variety of new digital tools have been developed since our searches were conducted. Although we aimed to overcome this by including protocols for future trials, the COVID-19 pandemic has seen the development of many new digital resources through necessity, which was not captured. An example of this is the Kidney Beam tool, developed and launched mid-pandemic to support PA virtually for people with kidney disease.^117^ The pandemic has accelerated the spread and adoption of digital resources^39^ and progressed the digital ambitions of the NHS.^16^ However, the adoption of digital tools by people with LTCs has traditionally been limited^39^ and it is currently unclear whether the pandemic will lead to longer-term usage of these tools. Consequently, it will be important to understand the impact of the pandemic on longer-term usage and the associated impact on NHS resources in future research.

Scoping reviews often support the development of focused research questions for future systematic reviews or other empirical studies. This review identified an increasing use of digital tools over the past decade when compared with a previous review,^29^ and included a considerable number of RCTs and protocols for RCTs. It would therefore be prudent to evaluate the effectiveness of these tools, alongside newly developed tools, using subgroup analyses to account for heterogeneity. Second, identification of key components of the interventions that successfully support the maintenance of PA for people with LTCs would be advantageous for future intervention development. As previously highlighted, an intervention component analysis^104^ approach may be most appropriate to achieve this. Finally, the continual focus on single conditions and younger age groups (both in published and planned studies) highlights the potential for a future-focused systematic review to investigate the factors influencing positive effects across these conditions. Such a review would support the development of effective interventions for people with multimorbidities.

## Conclusions

This scoping review aimed to identify and map the characteristics of existing and planned studies using digital tools for supporting people with LTCs to maintain PA. Our novel finding is the wealth of evidence across the 18 LTCs identified. Digital tools were commonly designed for people with CVD, type 2 diabetes mellitus and obesity and most often delivered via web browsers, with some interventions also combining wearable devices. PA outcomes were most often reported at 9 months or 3 months after the end of the intervention. Some studies clearly articulated the use of theories of behavioural change in the development of the interventions but greater reporting transparency is needed to maximise the synthesis of findings to establish effectiveness, future adoption and spread of digital tools.

## Supplemental Material

sj-docx-1-dhj-10.1177_20552076221089778 - Supplemental material for Digital tools to support the maintenance of physical activity in people with long-term conditions: A scoping review Click here for additional data file. Supplemental material, sj-docx-1-dhj-10.1177_20552076221089778 for Digital tools to support the maintenance of physical activity in people with long-term conditions: A scoping review by Paul Clarkson, Aoife Stephenson, Chloe Grimmett, Katherine Cook, Carol Clark, Paul E Muckelt, Philip O’Gorman, Zoe Saynor, Jo Adams, Maria Stokes and Suzanne McDonough in Digital Health

sj-docx-2-dhj-10.1177_20552076221089778 - Supplemental material for Digital tools to support the maintenance of physical activity in people with long-term conditions: A scoping review Click here for additional data file. Supplemental material, sj-docx-2-dhj-10.1177_20552076221089778 for Digital tools to support the maintenance of physical activity in people with long-term conditions: A scoping review by Paul Clarkson, Aoife Stephenson, Chloe Grimmett, Katherine Cook, Carol Clark, Paul E Muckelt, Philip O’Gorman, Zoe Saynor, Jo Adams, Maria Stokes and Suzanne McDonough in Digital Health
