# Supplementary material for: Digital tools to support the maintenance of physical activity in people with long-term conditions: A scoping review
Source: Digit Health. 2022 Apr 11;8:20552076221089778. doi: 10.1177/20552076221089778 (PMC9005829; doi:10.1177/20552076221089778)
Supplement: sj-docx-2-dhj-10.1177_20552076221089778 - Supplemental material for Digital tools to support the maintenance of physical activity in people with long-term conditions: A scoping review [file sj-docx-2-dhj-10.1177_20552076221089778.docx]

**Appendix A: Medline search strategy**

1. (MH Digital+)
2. TI ("Digital health" or "digital health intervention" or (E N1 health) or (M-Health) or (N-screen) or (N-device*) OR AB ("Digital health" or "digital health intervention" or (E-health) or (M-Health) or (N-screen) or (N-device*) OR (digital N1 therap*)
3. (MH internet+)
4. AB (intranet or internet or "internet delivered" or website* or "computer based" or software or online or "on-line" or net or web*
5. TI (intranet or internet or "internet delivered" or website* or "computer based" or software or online or "on-line" or net or web*
6. (MH electronic health+)
7. AB "e media" or emedia OR etrain* OR "e train"
8. TI "e media" or emedia OR etrain* OR "e train"
9. (MH mobile+)
10. ("mobile phone*" or smartphone* or smart* or cellphone* or "cell phone*" or "cellular phone*" or "hand held" or "digital device*" or "mobile technolog*" or "mobile device*" or "mobile health").ti.ab.
11. (MH electronic mail+)
12. ("text messag*" or sms or "short message service*" or email* or "electronic mail*"or MMS or "multimedia messag* service").ti.ab.
13. ("text messag*" or sms or "short message service*" or email* or "electronic mail*" or MMS or "multimedia messag* service").ti.ab.
14. (MH telemedicine+)
15. AB (telehealth or telerehabilitation or "pervasive technolog*" or "pervasive comput*" or "ubiquitous technolog*" or "ubiquitous comput*")
16. TI (telehealth or telerehabilitation or "pervasive technolog*" or "pervasive comput*" or "ubiquitous technolog*" or "ubiquitous comput*")
17. AB ((mobile or smart or phone or smartphone or "cell phone" or cellphone or tablet) N1 app*)
18. TI ((mobile or smart or phone or smartphone or "cell phone" or cellphone or tablet) N1 app*)
19. (MH actigraphy+)
20. TI (Fitness or activity or movement) N1 (track or monitor or sensor) or "just-in-time adaptive-intervention*" or lifelog* or "life log*" or "quantified self" or "self monitoring device*" or accelero* or pedometer or automated) OR AB (Fitness or activity or movement) N1 (track or monitor or sensor) or "just-in-time-adaptive-intervention*" or lifelog* or "life log*" or "quantified self" or "self monitoring device*" or accelero* or pedometer or automated)
21. TI wireless technology/ or AB wireless technology/
22. AB (wireless or wearable* or wristband* or "wrist band*" or wristworn or "wrist worn" or watch* or smartwatch* or "smart watch*")
23. TI (wireless or wearable* or wristband* or wrist band* or wristworn or "wrist worn" or watch* or smartwatch* or "smart watch*")
24. (MH gamification+)
25. AB (Exergaming or "exer-gaming" or virtual or gamercis* or "active video game*")
26. TI (Exergaming or "exer-gaming" or virtual or gamercis* or "active video game*")
27. AB Non-face-to-face
28. TI Non-face-to-face
29. TI Video* OR AB Video*
30. TI software OR AB software
31. AB "User computer interface"
32. TI "User computer interface"
33. TI Computer* OR AB Computer*
34. AB "Health technolog*"
35. TI "Health technolog*"
36. TI (Intervention or program*)
37. AB (Intervention or program*)
38. (MH Maintenance+)
39. TI Maintain*
40. AB Maintain*
41. TI Sustain*
42. AB Sustain*
43. TI Continu*
44. AB Continu*
45. TI Retain*
46. AB Retain*
47. TI Preserv*
48. AB Preserv*
49. TI Prolong*
50. AB Prolong*
51. TI "Follow up"
52. AB "Follow up"
53. TI "long-term"
54. AB "long-term"
55. TI Permanent
56. AB Permanent
57. TI Remain*
58. AB Remain*
59. TI (Adher* or compl* or concord*)
60. AB (Adher* or compl* or concord*)
61. TI ((Commit*) or N1 (device or intervention))
62. TI ((Commit*) or N1 (device or intervention))
63. AB ((Commit*) or N1 (device or intervention))
64. TI ((promot* or uptake* or encourage* or increase* or start* or adher* or sustain* or maintain*) N5 gym*))
65. AB ((promot* or uptake* or encourage* or increase* or start* or adher* or sustain* or maintain*) N5 gym*)
66. TI ((promot* or uptake* or encourage* or increase* or start* or adher* or sustain* or maintain*) N5 physical active*)
67. AB ((promot* or uptake* or encourage* or increase* or start* or adher* or sustain* or maintain*) N5 physical active*)
68. TI ((promot* or uptake* or encourage* or increase* or start* or adher* or sustain* or maintain*) N5 (circuit* or aqua*))
69. AB ((promot* or uptake* or encourage* or increase* or start* or adher* or sustain* or maintain*) N5 (circuit* or aqua*))
70. TI ((promot* or uptake* or encourage* or increase* or start* or adher* or sustain* or maintain*) N5 (exercise* or exertion or "keep fit" or "fitness class" or yoga or aerobic*))
71. AB ((promot* or uptake* or encourage* or increase* or start* or adher* or sustain* or maintain*) N5 (exercise* or exertion or "keep fit" or "fitness class" or yoga or aerobic*))
72. TI ((decreas* or reduc* or discourage*) N5 (sedentary or deskbound or physical* inactive*))
73. AB ((decreas* or reduc* or discourage*) N5 (sedentary or deskbound or physical* inactive*))
74. S38 OR S39 OR S40 OR S41 OR S42 OR S43 OR S44 OR S45 OR S46 OR S47 OR S48 OR S49 OR S50 OR S51 OR S52 OR S53 OR S54 OR S55 OR S56 OR S57 OR S58 OR S59 OR S60 OR S61 OR S62 OR S63 OR S64 OR S65 OR S66 OR S67 OR S68 OR S69 OR S70 OR S71 OR S72 OR S73
75. TI ("Physical* activ*" or "Physical* fitness" or "Physical exercise" or "physical exertion" or "physical endurance")
76. AB ("Physical* activ*" or "Physical* fitness" or "Physical exercise" or "physical exertion" or "physical endurance")
77. TI exercis*
78. AB exercis*
79. TI Fitness
80. AB Fitness
81. TI Sedentary
82. AB Sedentary
83. TI Sport*
84. AB Sport*
85. TI Walk*
86. AB Walk*
87. TI Run*
88. AB Run*
89. TI Jog*
90. AB Jog*
91. TI Pliates OR AB Pilates
92. TI Yoga OR AB yoga
93. ( TI ((cycle or cycling) N5 (school* or work or workplace or commut* or travel* or equipment or facility* or rack* or store* or storing or park* or friendly or infrastructure)) ) OR ( AB ((cycle or cycling) N5 (school* or work or workplace or commut* or travel* or equipment or facility* or rack* or store* or storing or park* or friendly or infrastructure)) )
94. TI Bicycle* OR AB Bicycle* OR bike* OR biking
95. ( TI (swim*) OR ( AB (swim*) )
96. TI rollerblad* OR AB rollerblad*
97. TI rollerskat* OR AB rollerskat*
98. TI "Strength training" OR AB "Strength training"
99. TI "resilience train*" OR AB resilience train*"
100. TI "weight lift*" OR AB "weight lift*"
101. TI (use N3 stair*) OR AB (use N3 stair*)
102. TI (graded N2 activit*) OR AB (graded N2 activit*)
103. (MH "long term condition"+)
104. TI chronic OR AB chronic
105. TI multimorbidit* OR "multi morbidit*" OR AB multimorbidit* OR "multi morbidit*"
106. S103 OR S104 OR S105
107. ( TI ("Atrial fibrillation" or "AF") ) OR ( AB ("Atrial fibrillation" or "AF") )
108. TI "Coronary heart disease" OR AB "Coronary heart disease"
109. TI "Cardiovascular disease" OR AB "Cardiovascular disease"
110. (MH "heart failure"+)
111. TI "heart failure" OR AB "heart failure"
112. TI "Congestive heart failure" OR AB "Congestive heart failure"
113. TI "Cardiac failure" OR AB "Cardiac failure"
114. ( TI ("Congestive cardiac failure" or "CCF") ) OR ( AB ("Congestive cardiac failure" or "CCF") )
115. ( TI ("Chronic heart failure" or "CHF") ) OR ( AB ("Chronic heart failure" or "CHF") )
116. TI "Chronic cardiac failure" OR AB "Chronic cardiac failure"
117. TI "Ventricular dysfunction" OR AB "Ventricular dysfunction"
118. TI Hypertension OR AB Hypertension
119. TI "High blood pressure" OR AB "High blood pressure"
120. TI "Peripheral arterial disease" OR AB "Peripheral arterial disease"
121. TI Stroke OR AB Stroke
122. ( TI (ischaemic or ischemic) ) OR ( AB (ischaemic or ischemic) )
123. ( TI (haemorrhage* or haemorrhage*) ) OR ( AB (haemorrhage* or haemorrhage*) )
124. ( TI "Cerebrovascular attack" or "CVA") ) OR ( AB "Cerebrovascular attack" or "CVA") )
125. (TI "Transient ischaemic attack" or "TIA") ) OR ( AB "Transient ischaemic attack" or "TIA"))
126. S121 OR S122 OR S123 OR S124 OR S125
127. TI asthma* OR AB asthma*
128. TI Wheez* OR AB Wheez*
129. TI Bronchoconstrict* OR AB Bronchoconstrict*
130. TI Antiasthma* OR AB Antiasthma*
131. TI "Anti-asthma*" OR AB "Anti-asthma*"
132. TI "Respiratory sounds" OR AB "Respiratory sounds"
133. TI "Bronchial hyperreactivity*" OR AB "Bronchial hyperreactivity*"
134. TI Bronchospas* OR AB Bronchospas*
135. ( TI (Obstruct* N3 (pulmonary or lung* or airway* or airflow* or bronch* or respirat*)) ) OR ( AB (Obstruct* N3 (pulmonary or lung* or airway* or airflow* or bronch* or respirat*)) )
136. TI Chronic* N3 bronchiti* OR AB Chronic* N3 bronchiti*
137. ( TI ("Chronic obstructive pulmonary disease" or "COPD") ) OR ( AB ("Chronic obstructive pulmonary disease" or "COPD") )
138. TI "Lung disease*" OR AB "Lung disease*"
139. TI Emphysema* OR AB Emphysema*
140. ( TI ("Chronic obstructive airway disease" or "COAD") ) OR ( AB ("Chronic obstructive airway disease" or "COAD") )
141. ( TI ("Chronic airflow limitation" or "CAL") ) OR ( AB ("Chronic airflow limitation" or "CAL") )
142. ( TI ("Chronic obstructive lung disease" or "COLD") ) OR ( AB ("Chronic obstructive lung disease" or "COLD") )
143. ( TI ("Chronic obstructive bronchopulmonary disease" or "COBD") ) OR ( AB ("Chronic obstructive bronchopulmonary disease" or "COBD") )
144. ( TI ("Acute exacerbation of chronic bronchitis" or "AECB") ) OR ( AB ("Acute exacerbation of chronic bronchitis" or "AECB") )
145. ( TI ((bronchial* or respiratory or airway* or lung*) N3 (hypersensitive* or hyperreactiv* or allerg* or insufficiency)) ) OR ( AB ((bronchial* or respiratory or airway* or lung*) N3 (hypersensitive* or hyperreactiv* or allerg* or insufficiency)) )
146. ( TI Bronch* N3 (constrict* or spas*) ) OR ( AB Bronch* N3 (constrict* or spas*) )
147. TI "Diabetes mellitus" OR AB "Diabetes mellitus"
148. (MH "Diabetes mellitus, type1"+)
149. (MH "Diabetes mellitus, type2"+)
150. TI "Insulin resistance" OR AB "Insulin resistance"
151. TI "Diabetic ketoacidosis" OR AB "Diabetic ketoacidosis"
152. TI (diabet* or dm) N5 (typ* N3 (one or 1 or I)) OR AB (diabet* or dm) N5 (typ* N3 (one or 1 or I))
153. TI (diabet* or dm) N5 (typ* N3 (two or 2 or II)) OR AB (diabet* or dm) N5 (typ* N3 (two or 2 or II))
154. TI (Insulin or noninsulin or non-insulin) N2 (resistan* or depend*) OR AB (Insulin or noninsulin or non-insulin) N2 (resistan* or depend*)
155. TI Diabet* OR AB Diabet*
156. TI ("Non insulin dependent diabetes mellitus" or NIDDM) OR AB ("Non insulin dependent diabetes mellitus" or NIDDM)
157. TI ("Insulin dependent diabetes mellitus" or IDDM) OR AB ("Insulin dependent diabetes mellitus" or IDDM)
158. TI Glucose N1 (tolerance or intolerance) OR AB Glucose N1 (tolerance or intolerance)
159. S147 OR S148 OR S149 OR S150 OR S151 OR S152 OR S153 OR S154 OR S155 OR S156 OR S157 OR S158
160. (MH Dementia+)
161. TI Alzheimers OR AB Alzheimers
162. TI "Cognitive impairment" OR AB "Cognitive impairment"
163. TI "Memory loss" OR AB "Memory loss"
164. TI "Vascular dementia" OR AB "Vascular dementia"
165. S160 OR S161 OR S162 OR S163 OR S164
166. (MH Depression+)
167. TI Depress* OR AB Depress*
168. S166 OR S167
169. TI Epilep* OR AB Epilep*
170. (MH "Mental health"+)
171. (MH "Mental health"+)
172. TI Osteoporo* OR AB Osteoporo*
173. TI Arthriti* OR AB Arthriti*
174. ( TI (Osteoarthritis or OA) ) OR ( AB (Osteoarthritis or OA) )
175. ( TI (Rheumatoid arthritis or RA) ) OR ( AB (Rheumatoid arthritis or RA) )
176. S172 OR S173 OR S174 OR S175
177. S127 OR S128 OR S129 OR S130 OR S131 OR S132 OR S133 OR S134 OR S135 OR S136 OR S137 OR S138 OR S139 OR S140 OR S141 OR S142 OR S143 OR S144 OR S145 OR S146
178. TI ((Fitness or activity or movement) N1 (track or monitor or sensor)) or "just in time adaptive intervention*" or lifelog* or "life log*" or "quantified self" or "self monitoring device*" or accelero* or pedometer) OR AB ((Fitness or activity or movement) N1 (track or monitor or sensor)) or "just in time adaptive intervention*" or lifelog* or "life log*" or "quantified self" or "self monitoring device*" or accelero* or pedometer)
179. TI ( wireless OR wearable* OR wristband* OR "wrist band*" OR wristworn OR "wrist worn" OR watch* OR smartwatch* OR "smart watch*" ) OR AB ( wireless OR wearable* OR wristband* OR "wrist band*" OR wristworn OR "wrist worn" OR watch* OR smartwatch* OR "smart watch*" )
180. AB ( "text messag*" OR sms OR "short message service*" OR email* OR "electronic mail*" OR MMS OR "multimedia messag* service" ) OR TI ( "text messag*" OR sms OR "short message service*" OR email* OR "electronic mail*" OR MMS OR "multimedia messag* service" )
181. S1 OR S2 OR S3 OR S4 OR S5 OR S6 OR S7 OR S8 OR S9 OR S11 OR S14 OR S15 OR S16 OR S17 OR S18 OR S19 OR S20 OR S21 OR S24 OR S25 OR S26 OR S27 OR S28 OR S29 OR S30 OR S31 OR S32 OR S33 OR S34 OR S35 OR S179 OR S180
182. AB ( ((promot* or uptake* or encourage* or increase* or start* or adher* or sustain* or maintain* OR prolong* OR "long term" OR permanent OR "Follow up" OR comply OR complian* OR concord*) N5 ("fitness class" or yoga or aerobic* OR "physical* activ*" OR circuit* OR aqua* OR exercis* OR exertion OR "keep fit")) ) OR TI ( ((promot* or uptake* or encourage* or increase* or start* or adher* or sustain* or maintain* OR prolong* OR "long term" OR permanent OR "Follow up" OR comply OR complian* OR concord*) N5 ("fitness class" or yoga or aerobic* OR "physical* activ*" OR circuit* OR aqua* OR exercis* OR exertion OR "keep fit")) )
183. AB ( (decreas* or reduc* or discourage*) N5 (sedentary or deskbound OR "desk bound"or "physical* inactive"*) ) OR TI ( (decreas* or reduc* or discourage*) N5 (sedentary or deskbound OR "desk bound"or "physical* inactive"*) )
184. S75 OR S76 OR S77 OR S78 OR S79 OR S80 OR S81 OR S82 OR S83 OR S84 OR S85 OR S86 OR S87 OR S88 OR S89 OR S90 OR S91 OR S92 OR S93 OR S94 OR S95 OR S96 OR S97 OR S98 OR S99 OR S100 OR S101 OR S102
185. S182 OR S183 OR S178
186. S181 AND S184 AND S185
187. S106 AND S186
188. S106 OR S107 OR S108 OR S109 OR S110 OR S111 OR S112 OR S113 OR S114 OR S115 OR S116 OR S117 OR S118 OR S119 OR S120 OR S126 OR S127 OR S128 OR S129 OR S130 OR S131 OR S132 OR S133 OR S134 OR S135 OR S136 OR S137 OR S138 OR S139 OR S140 OR S141 OR S142 OR S143 OR S144 OR S145 OR S146 OR S159 OR S165 OR S168 OR S169 OR S170 OR S171 OR S176
189. S186 AND S188
190. TI ( "chronic Kidney Disease" OR CKD ) OR AB ( "chronic Kidney Disease" OR CKD )
191. TI (obes* OR overweight) OR AB (obes* OR overweight)
192. S188 OR S190 OR S191
193. S189 AND S192

Limits: 01/01/2009 – 31/12/2019 / All adults: 19+ / Language: English

**Appendix B**

**Digital review criteria**

1. Exclude: Interventions where ‘digital’ is not a major component (more than 50% of the intervention),
2. exclude if only intervention is automated prompts (text or voice) for responding to the research task (e.g. reminders to complete assessment or questionnaire),
3. exclude if more face to face/in-person (home visits) than digital, i.e. exclude if 5 home visits and 2 telephone calls,
4. exclude if the only telerehabilitation component is a phone line/helpline for participants to call if required,
5. exclude if main purpose is to provide education or support to health care provider rather than social support.

**Appendix C**

**Charting categories**

| Study citation |
| --- |
| Study design |
| Country and number of sites |
| Setting |
| Study focus |
| Intervention description |
| Inclusion/exclusion criteria |
| Number of participants (Control and Intervention group, is applicable) |
| Age of participants (Mean/Standard deviation) (Control and Intervention group, is applicable) |
| Gender of participants (N/%) (Control and Intervention group, is applicable) |
| Primary long-term condition |
| Additional long-term condition/comorbidity |
| Theoretical underpinning (Primary) |
| Other theoretical underpinning |
| Use of the BCT Taxonomy (Michie et al 2013) |
| Length of follow-up |
| Access to intervention during maintenance period |
| How was effectiveness of PA measured? (if appropriate) (Subjective or Objective) |
| Effectiveness of the intervention (Which methods were used and what were the results) |
| Author reported limitations |
| Experiences of intervention use for maintenance of PA |
| Barriers and facilitators to maintaining PA |

**Summary tables**

**Study characteristics**

| **Study** | **Design** | **Study description** | **Primary long-term condition** | **Location of study** | **Gender of overall study participants**  N/% Female | **Sample size (Included at baseline)** | **N (Digital intervention group at baseline)** | **Age of digital intervention group at baseline (unless stated)** Mean (SD) |
| --- | --- | --- | --- | --- | --- | --- | --- | --- |
| **Randomised controlled trials / Experimental studies** | | | | | | | | |
| Lubans et al 2009 (49) | RCT | Assessor blinded RCT with mediation analysis | Obesity | Australia | NR | 65 | 34 | 37.5 (10.4) |
| Hurkmans et al 2010 (50) | RCT | Follow up data from an RCT | Rheumatoid Arthritis | The Netherlands | 83 (75.4%) | 160 | 82 | Median 49.5 (12.9 IQR) (intervention group only) |
| Lorig et al 2010 (51) | RCT | RCT | Type-2 Diabetes Mellitus | USA | 556 (73%) at baseline | 761 | 491 | 54.3 |
| Reid et al 2012 (52) | RCT | RCT to validate the programme | Cardiovascular Disease | Canada | 35 (15.7%) | 223 | 115 | 56.7 (9) |
| Bossen et al 2013 (53) | RCT | 2 arm RCT | Osteoarthritis | The Netherlands | 129 (64.8%) | 199 | 100 | 61 (5.9) |
| Ström et al 2013 (54) | RCT | RCT | Depression | Sweden | 40 (83.3%) | 48 | 24 | 48.8 (12.7) |
| Devi et al 2014 (55) | RCT | RCT with 2 parallel groups | Cardiovascular Disease | England | 24 (25.5%) | 94 | 48 | 66.27 (8.35) |
| Jennings et al 2014 (56) | RCT | RCT | Type-2 Diabetes Mellitus | Australia | 189 (47.6%) | 397 | 195 | 58.21 (10.6) |
| Verwey et al 2014 (68)  Van der Weegen et al 2015 (57) | Protocol  RCT | Protocol for RCT  Cluster RCT | COPD  Type-2 Diabetes Mellitus or COPD | The Netherlands | 102 (51.3%) | 240 (Groups 1,2,3)  199 (Groups 1,2,3) | 80  65 | 57.5 (7) |
| Olson et al 2015 (58) | RCT | 2-arm RCT (control group of interest) | Type-2 Diabetes Mellitus | USA | 75 (64.7%) | 116 | 62 (Control) | 62.4 (6.9) |
| Vorrink et al 2016 (59) | RCT | Multicentre investigator blinded RCT | COPD | The Netherlands | 79 (50.3%) | 183 | 102 | 62 (9) |
| Thorup et al 2016 (60) | RCT | Intervention arm of an RCT | Cardiovascular Disease | Denmark | 13 (20%) | 151 | 75 | 62.8 (11.5) |
| Givon et al 2016 (61) | RCT | single-blind randomized controlled trial | Stroke | Israel | 19 (40.4%) | 47 | 23 | 56.7 (9.3) |
| Yang et al 2017 (62) | RCT | crossover design RCT | Obesity | Taiwan | 16 (61.5%)  Group A (maintenance) only | 53 | 26 | 33.9 (10) |
| Lari et al 2018 (67) | Quasi-experimental study | Quasi -experimental study | Type-2 Diabetes Mellitus | Iran | 34 (46.6%) | 80 | 40 | 46.1 (9.14) |
| Alonso-Dominguez et al 2017 (69)  2019 (63) | Protocol  RCT | Study protocol for a randomised controlled trial  Randomised clinical trial | Type-2 Diabetes Mellitus | Spain | 93 (45.6%) | 200  204 | 100  102 | Median 60.8 (IQR: 7.8) |
| Dor-Haim et al 2019 (64) | RCT | RCT (study ongoing, n=20 recruited – preliminary results) | Cardiovascular Disease | Israel | NR | 20 | Unclear | NR |
| Harrison et al 2019 (70)  Patel et al 2019 (65) | Protocol  RCT | Protocol  4-arm RCT | Obesity | USA | 175 (29.1%) | 602  602 | 451 | 39.4 (10.1)  38.3 (10)  38.7 (10) |
| Avila et al 2019 (66) | RCT | 3-arm RCT | Cardiovascular Disease | Belgium | 8 (10%) | 90 | 60 | 62 (7.4) Centre-based  62.2 (7.1) Home-based |
| **Protocols** | | | | | | | | |
| Barry et al 2011 (71) | Protocol | 4-arm RCT study design | Obesity | USA | - | 197 | 98 | - |
| Kloek et al 2014 (72) | Protocol | Protocol for RCT | Osteoarthritis | The Netherlands | - | 200 | 100 | - |
| Fife-Schaw et al 2014 (73) | Protocol | 4-arm RCT protocol | Cardiovascular Disease | UK | - | 2000 (4 x 500) | 1000 (2 x 500) | - |
| Banos et al 2015 (74) | Protocol | Protocol for an RCT (trial underway) | Obesity | Spain | - | 100 | 50 | - |
| Jaarsma et al 2015 (75) | Protocol | Multicentre RCT rationale, design and methodology | Cardiovascular Disease | Sweden, Italy, Israel and The Netherlands | - | 600 | 300 | - |
| Brouwers et al 2017 (76) | Protocol | Monocentre 2 arm RCT protocol | Cardiovascular Disease | The Netherlands | - | 300 | 150 | - |
| Ingram et al 2018 (77) | Protocol | Protocol for RCT | Obesity | UK | - | 413 | 207 | - |
| Cox et al 2018 (78) | Protocol | protocol for RCT: assessor-blinded equivalence trial | COPD | Australia | - | 142 | 64 | - |
| Bonn et al 2018 (79) | Protocol | 2 arm RCT protocol | Type-2 Diabetes Mellitus | Sweden | - | 200 | 100 | - |
| Volders et al 2019 (80) | Protocol | Study protocol for parallel 2 group cluster RCT | At least one chronic condition. Mixed** | The Netherlands | - | 540 | 270 | - |
| Sharma et al 2019 (81) | Protocol | Rationale and design for RCT | Cardiovascular Disease | USA | - | 400 | 200 | - |
| **Pilot/Feasibility Studies** | | | | | | | | |
| Jones et al 2016 (82) | Feasibility | Single-group design - feasibility/acceptability study | Stroke | Australia | 14 (58.3%)  (Includes stroke and TBI population) | - | 24 | 51.13 (16.52)  (Includes stroke and TBI population) |
| Vorderstrasse et al 2017 (83) | Pilot study | Pilot study for RCT | Type-2 Diabetes Mellitus | USA | 129 (62%) | 208 | Unclear (Target 150 in each group) | 59 (NR) |
| Hawkins et al 2019 (84) | RCT | Feasibility RCT with process evaluation and economic analysis | No single LTC reported | Wales | 101 (64.7%) | 156 | 88 | 55.1 (17.6) |
| **Other studies** | | | | | | | | |
| Barnason et al 2016 (85) |  | Single group correlational design (relationship between self-efficacy and exercise in the “parent” study) | Cardiovascular Disease | USA | NR | - | 39 | NR |
| Verwey et al 2016* (86) | Mixed method study | Mixed method process evaluation study | Type-2 Diabetes Mellitus or COPD | The Netherlands | 62 (54.9%) | - | 131 (Groups 1,2)  20 nurses | 58 (7.7)  43.4 Nurses |

RCT, Randomised controlled trial; USA, United States of America; LTC, Long-term condition; COPD, Chronic Obstructive Pulmonary Disease

**Characteristics of the digital tools**

| **Author** | **Intervention Description** | **Hardware of the digital device** | **Intervention components including non-digital** | **Type of Digital Device Used (WHO)** | **Length of intervention** | **Longest length of maintenance period** | **Access to device/ intervention during maintenance period** | **PA measure** | **Objective or Subjective measure** |
| --- | --- | --- | --- | --- | --- | --- | --- | --- | --- |
| *Vorrink et al 2016* | Real time physical activity tracking with automated persuasive messages from physios.  Goal setting - monitored and updated by physios online. | App-based | Participants used a smartphone app to help maintain physical activity following pulmonary rehabilitation. Physios also had access to website for monitoring. | Personal health tracking | 6 months | 6 months | Based on Figure 1. It appears that the smartphone was returned at 6 months, based on the list of drop out reasons at T6 "Upset that smartphone had to be returned" | Device based | SenseWear Armband & Mobile device used in intervention (steps) |
| *Thorup et al 2016* | Personally tailored cardiac telerehabilitation programme with specific aim to increase step count | Telerehabilitation with access to a wearable device | Nurses prescribed individual rehab programme with daily step goals, linked to different settings (health care centre, hospital, call centre) | Telerehabilitation/ Telemedicine  Targeted client communication with personal health tracking | 3 months | 9 months | Access to online health information and the step counter only | Device based | Fitbit Step Counter |
| *Lorig et al 2010* | Internet based self-management programme is an asynchronous, 6-week, self- management internet-based tool consisting of 6 weekly sessions. One group had access to email reinforcement messages | Web-based | Participants logged on individually to the sessions, which were available for the entire week. Other resources online included a learning centre, discussion centre, my tools, post office, help and a free phone line. Participants also received a copy of the book,  Living a Healthy Life with Chronic Condition. Facilitators assisted participants with use of the programme | Targeted Client Communication | 6 weeks | 16 months, 2 weeks | IDSMP consisted of 6 weekly sessions, so unlikely to have contact during maintenance period | Participant report | Physical activity scale |
| *Jones et al 2016* | A remote delivered self-management programme for people with acquired brain injury (ABI) | Web-based | Six online modules delivered over the intervention period. No specific physical activity intervention - aimed to empower individuals with the knowledge and skills required to build and manage their own physical activity | Targeted Client Communication | 8 weeks | 3 months | Only brief admin contact | Device based | ActivPAL3 and Actigraph |
| *Hurkmans et al 2010* | Internet-based physical activity intervention | Web-based | Participants performed strengthening, ROM and cycling exercises on a loaned cycle ergometer in their home. The program had to be performed 5 times a week on 5 separate days. Every week the patients sent back a completed program schedule by e‐mail, and subsequently a new schedule was put on the individual's personal Web page. Patients received weekly, individual distant supervision by e‐mail from 2 experienced physical therapists. | Targeted Client Communication | 12 months | 12 months | Figure 1 flow diagram states ' Follow-up: no intervention' | Participant report | ' 2 Questionnaires' - how many days active @moderate & vigorous |
| *Lari et al 2018* | SMS distinct training programme | Phone-based text messages | The intervention lasted 12 days whereby 2-3 SMS messages were sent to the intervention group each day based on health promotion modelling. The messages included information on how to be active, control blood sugar, sports rules, physical activity and blood sugar levels, complications of diabetes, barriers to physical activity and how to ask for support from friends and family. After the 12 days, participants could ask researchers questions. | Targeted Client Communication | 12 days | 3 months | Reduced amount of text messages received (2 per week) | Participant report | 7-day recall during semi-structured interview (extracted FITT of exercise) |
| *Jaarsma et al 2015* | Structured access to a Wii game computer to increase daily activity and for rehabilitation purposes | Gaming Device | All patients were advised to be active for 30 minutes per day, but advice will be adapted to each participant. Those in the Wii group will be given an intro lesson (1 hour) on how to use the Wii and the Wii will be installed at patient's home. They will be asked to try play for 30 minutes per day. Patients will receive regular treatment and information about rehabilitation and daily physical activity. The patients will also receive activity advice and be followed by telephone at 2, 4, 8 and 12 weeks to talk about their experiences, receive motivational support or to sort out any issues. | Gamification/ Exergaming  Untargeted client communication | 3 months | 9 months | Unclear whether they have access to the Wii for the whole 12-month period, but the addition of telephone support is only present in the first 3 months. | Device based | Actigraph Accelerometer |
| *Jennings et al 2014* | Fully automated web-based programme | Web-Based | The programme utilises a self-management approach. The self-management approach aims build skills and abilities to initiate and maintain health-related behaviour change. In addition to the website, participants in the intervention group were also distributed a weekly email reminder, the content of which changed weekly, but always contained a link to the intervention website. Website included logbooks, goals, email reminders. A pedometer was also provided for motivational and self-monitoring purposes | Targeted Client Communication with personal health tracking | 12 weeks | 24 weeks | Website remained accessible but no further updates were provided. Pedometers kept after end of intervention period | Participant report | IPAQ |
| *Hawkins et al 2019* | Activity monitor (MyWellnessKey) and web platform (MyWellnesCloud) and smartphone application | MyWellnessKey, MyWellnesCloud, smartphone application  Wearable with connection to web-based platform | Exercise Referral Scheme delivered by exercise professionals for 16 weeks. Access to digital tools from 4 weeks into the scheme. | Personal health tracking | 16 weeks | 8 months | They had access to the MyWellnessKey during the follow up period if they wanted to. Unclear | Device based | Actigraph Accelerometer |
| *Dor-Haim et al 2019* | Digital home-based cardiac rehabilitation | Smart garment incorporating ECG monitoring, cloud-based artificial intelligence server for monitoring of vital signs, transmitted to a smartphone/ tablet app for user and for professional review  Wearable with connection to mobile app | Participants attended the cardiac rehabilitation site biweekly for first 6 weeks | Personal health tracking | 3 months | 9 months | Appears as there is no access during maintenance period | Device based | Fitbit Step Counter |
| *Devi et al 2014* | Web-based platform (Activate your heart) to log physical activity, goal setting and information to support physical activity and other cardiac risk factors | Web-based | Feedback on goals provided. Participants could request support from cardiac nurse through the platform | Personal health tracking, Targeted client communication | 6 weeks | 4.5 months | Absence of continued access to the site or any ongoing support | Device based | SenseWear Armband |
| *Barnason et al 2016* | Telehealth cardiac rehabilitation (CR) intervention | Telerehabilitation | Treatment group received intervention plus cardiac rehab programme as part of post-cardiac revascularisation | Telerehabilitation/ Telemedicine  Untargeted client communication | From potential linked paper: 12-week CR with post assessments at month 4 | 3 months | Linked paper suggests no access during maintenance period | Device based | Actigraph Accelerometer |
| Harrison et al 2019  Patel et al 2019 | Wearable device used to measure steps/day using support, competitive and collaborative gamification arms | Wearable device (Withings/ Nokia Steel) + gamification of intervention | 4-week ramp up period to set steps goal, then goal to maintain over following 20 weeks and 12-week maintenance period. Points based goals system with three levels that participants could move between. 3 arms to intervention group (support - friend/family member sent info on progress), Competitive (group of 3 participants with leadership board sent each via message each week), Collaborative (group of 3 participants, 1 of 3 represents group each day to meet goal). Phone call contact at 8/16 weeks if needed. | Gamification/ Exergaming  Personal health tracking, Targeted client communication | 24 weeks | 12 weeks | Unclear | Device based | Device used for the intervention |
| *Brouwers 2017* | Web platform, sensors (HR monitor, accelerometer, video consultations) | Telerehabilitation, web-based, sensors | First 6-weeks supervised group-based exercise activities. After 6 weeks participant uploads activity data from sensor to web platform and receives video consultation with physio (using motivational interviewing techniques) up to 12-weeks. 9 months of telecoaching followed +more for those who needed it). | Telerehabilitation/ Telemedicine  Targeted client communication | 3 months | 9 months (access to telecoaching) | Appears to have access for duration of follow-up but the system is not updated/adjusted to target new goals. Patients can use telecoaching on demand during 9-month maintenance | Device based | Actigraph accelerometer and Polar chest strap |
| *Bossen 2013* | Automated web-based intervention with automatic text messaging and email  Goal setting, fixed time point objectives for physical activity | Web-based | 9-week independently conducted with favourite recreational activity gradually increased during the programme. Self-test to develop short term goals. Tailored modules automatically generated. Automated web-based intervention with text messaging and email, without additional support | Targeted Client Communication | 9 weeks | 9 months, 3 weeks | No - Each module available for one week only | Device based and participant report | Actigraph (provided to a subgroup only) and  PA scale for elderly |
| *Bonn 2018* | Mobile app based (DiaCert)  Goal setting - If achieved user receives a positive message. | Mobile app with access to digital platform for carers | Standard care + app in intervention group (app introduced at first meeting) Step goal set between participant and research team based on usual activity level and revised each 2 weeks. | Personal health tracking, Targeted client communication | 12 weeks | 9 months | Participants don't have access for the 3 months of F/U but then get access after this for the remaining 6 months F/U | Device based | Actigraph Accelerometer |
| *Barry 2011* | Sensewear arm band activity monitoring system for self-monitoring | Sensewear arm band, and platform + web account to 'personalised weight management solutions'  Wearable with connection to web-based platform | Sensewear intervention group received minimal contact from study staff, other than initial training. Additional groups: sensewear + self-directed group-based weight loss programme + 6x FU phone calls; self-directed group-based weight loss programme only; standard care group (weight loss manual only) | Personal health tracking | 4 months | 5 months | 6 follow up telephone calls during maintenance period. Unclear whether digital tool used during this period | Device based | SenseWear Armband |
| *Fife-Schaw 2014* | 12-week access to a self-management website with and without sports-based exercise referral from GP | Web-based | Web-based information resource and physical activity programme. Exercise referral scheme includes a choice of supervised sport activities | Untargeted client communication | 12 weeks | 9 months | Regular interactive reminders during follow-up period | Participant report | IPAQ and the Self-Report Walking and Exercise Tables |
| *Avila 2019* | Home-based exercise with telemonitored guidance/supervision from physios after cardiac rehabilitation | Telemonitoring - heart rate, exercise intensity and data upload (via Garmin monitor and online application) | Home-based: 3 supervised sessions with physio, then individualised exercise programme. Weekly feedback provided via phone/email | Telerehabilitation/ Telemedicine  Targeted client communication | 3 months | 9 months | No contact or feedback provided by the research group after 3 months | Device based | SenseWear Armband |
| *Olson 2015* | Control group: online education course | Web-based | Exercise consultation after study conclusion for web-based control group.  Study intervention group: Group-based walking/workshops | Untargeted client communication | 8 weeks | 4 months | No access during maintenance period | Device based | Actigraph Accelerometer |
| *Alonso-Domínguez 2017*  *Alonso-Domínguez 2019* | Mobile app-based - healthy walks and nutrition workshop in groups | App-based | Training in use of app during same week as intervention. Individual appointments to provide advice on physical activity. Use of the app is group-based and used to assess whether an individual's habits agree with recommendations for physical activity. | Targeted Client Communication and personal health tracking | 3 months | 9 months | No access during maintenance period | Device based and participant report | Omron digital pedometer (tri-axial) and IPAQ |
| *Verwey 2014*  *Verwey 2016*  *van der Weegen 2015* | It's life' tool - digital intervention and support group as part of a self-management support programme.  Goal setting, monitoring | Accelerometer, mobile app, server/web application | Consultation with practice nurse at start and at intervals throughout use of the intervention. Users receive feedback through the mobile app. Nurses monitor physical activity on web application. | Targeted Client Communication and personal health tracking | 24 weeks | 3 months | No indication of further practice nurse contact or use of the intervention after 4-6 months | Device based and participant report | AM300 activity monitor and SQUASH questionnaire |
| *Kloek 2014* | Web-based physical activity 3-month programme  Goal setting | Web-based | exercise sessions delivered online with maximum of 5 face-to-face sessions. Graded activities to meet goals  Intervention also an information resource | Targeted Client Communication | 3 months | 9 months | No access during maintenance period | Device based and participant report | Actigraph Accelerometer and SQUASH questionnaire |
| *Ingram 2018* | ‘eCoachER' web-based tool for use alongside an exercise referral scheme | Web-based, Pedometer | Core elements of the intervention are the '7 steps to health' to consider the benefits of physical activity, to utilise support from scheme practitioners, social networks and the internet, goal setting, self-monitoring with a pedometer/ uploading to web tool, sustainable physical activity in daily life, reducing the impact of setbacks. | Targeted Client Communication, Personal health tracking | 3 months typically | 9 months (option to continue exercise referral programme for the full 12 months) | Participants have the option to continue the exercise referral programme for the full duration. Unclear | Device based and participant report | GENEActiv original accelerometer and unclear |
| *Ström 2013* | 9 modules online self-help programme with pedometer  Includes action planning | Web-based, Pedometer | Modules include weekly exercise planning (goal setting) and questions for users to complete. Pedometer used to monitor activity for motivational purposes (no data collected). Written feedback based on motivational interviewing from therapists | Targeted Client Communication, Personal health tracking | 9 weeks | 3 months and 3 weeks | Participants in intervention group had access to the pedometer and could use it if they wanted to but was not monitored if they used it or not. Unclear whether there was continued access to the internet-based system after the 9-week active intervention. | Participant report | IPAQ |
| *Vorderstrasse 2017* | Learning in virtual environment (LIVE) intervention to provide diabetes self-management training | Web-based with gamification | Computer-based virtual environment with interactions as an avatar. Includes group information sharing, social networking, gamification aspects, ability to visit different locations. | Gamification/ Exergaming  Targeted client communication | 12 months | 6 months | Unclear | Device based and participant report | Fitbit step counter and Stanford brief activities scale & SDSCA sub scale for PA |
| *Reid 2012* | Web-based 'CardioFit' system tutorials | Web-based | Participants receive initial assessment and have a personalised plan (goal setting) set up while still in hospital using the CardioFit system. Participants receive web tutorials. Users could upload daily physical activity on site, ask questions and receive motivational emails on their progress | Targeted Client Communication | 6 months | 6 months | Participants have access to website during FU 6 months, but without the tutorials. Participants still receive feedback based on PA programme to date based on steps logged by participant and receive a new programme every 6 weeks until week 50 | Device based and participant report | YAMAX Pedometer and Godin Leisure-Time Exercise Questionnaire |
| *Volders 2019* | ‘Active Plus' personalised physical activity advice web-based intervention | Web-based | Personalised physical advice online and in print based on answers to questionnaires, opportunity to contact other participants | Targeted Client Communication with client-to-client communication option | 4 months | 8 months | Access to website for some of the maintenance period but (until 6 months) but not afterwards | Device based and participant report | Actigraph Accelerometer and SQUASH questionnaire |
| *Lubans 2009* | Self-Help, Exercise and Diet using Information Technology Programme ('SHED-IT') internet-based weight loss intervention with access to online support. | Web-based | Access to website - www.calorieking.com.auonline support/ feedback/goal setting based on completion of diaries by participants, and one face-to-face session, participants able to record levels of daily physical activity and post questions on the website. | Targeted Client Communication with personal health tracking | 3 months | 3 months | Access to the website but no online feedback | Device based | YAMAX Pedometer |
| *Givon 2016* | Video games intervention as part of occupational therapy (OT) activities focused on upper limb movement, balance and walking | Video game consoles/games (Microsoft Xbox Kinect, Sony PlayStation 2 Eyetoy, Sony PlayStation 3 MOVE, Nintendo Wii Fit and the SeeMe VR system) | Intervention started with warm up (walking) using the Wii console, before dividing participants to different consoles in pairs and then rotated to other consoles. Guidance/ supervision provided by OTs. When not using console, participants encouraged to respond to others by clapping/ standing up. | Gamification/ Exergaming  Untargeted client communication | 3 months | 3 months | No access during maintenance period | Device based | Acticial acclerometer |
| *Yang 2017* | Activity promotion system, including app, with wearable device and web-based feedback system | Web-based, Wearable | Physical activity monitoring via wearable that can be uploaded to web-based system and compared to peer group. Health recommendations/information on site, reminder messages, counselling support and feedback, goal setting online and email. | Targeted Client Communication, Personal health tracking | 3 months | 3 months | No access during maintenance period | Device based | PA sensor developed by the research team (Triaxial digital accelerometer - Lin et al 2012 cited) |
| *Sharma et al 2019* | Wearable device for personalised physical activity tracking and feedback via short messaging service (SMS) | Withings Go activity tracker / SMS | Step count recorded daily using activity tracker and used to summarise performance, receive personalised feedback and set goals for the following week via SMS  Additional web-based application (Duke Pillbox) used by participants in study for medications adherence | Targeted Client Communication | 3 months | 3 months | Access to the activity tracker only during maintenance period with some interaction with study investigators via phone call/in-person | Device based | Device used for the intervention |
| *Cox 2018* | Group-based pulmonary rehabilitation exercises delivered at home via telerehabilitation | Telerehabilitation delivered via iPad/Zoom to healthcare professional Pulse oximeter. | Initial set up by physio during a home visit before telerehabilitation delivered via video call using Zoom. Participant uses exercise bike to complete exercises and has pulse oximeter which can be monitored by physio. Participants are able to see and interact with other group members.  Participants also receive specific self-management information through a booklet. Goal setting included. | Telerehabilitation / Telemedicine  Targeted client communication | 8 weeks | 10 months | Offer of participation in supervised exercise maintenance programme after 8 weeks based on pulmonary rehabilitation guidelines. Monthly follow-up phone calls also. | Device based | GENEActiv original accelerometer |
| *Banos 2015* | Living better' internet-based intervention made up of 9 modules to change physical activity gradually | Web-based | Modules delivered once per week for 5 weeks and then once every two weeks include: education on physical activity, strategies to reduce barriers, goal setting, problem solving, improving body image and preventing relapse. Participants receive welcome email and another motivational email after each module. Contact from research team if participant stops using intervention for 2 weeks. | Targeted Client Communication | 3 months | 9 months | Likely no access during the maintenance period | Participant report | IPAQ |

App, Application; SMS, Short Messaging Service; IPAQ, International Physical Activity Questionnaire; ECG, Electrocardiogram; CR, Cardiac rehabilitation; FU, Follow up; SQUASH, Short Questionnaire to Assess physical activity; VR, Virtual Reality

**Appendix C: Theoretical underpinning**

| **Study** | **Theory- based intervention** | **Theory** | **Unclear use of theory** | **Theory** |
| --- | --- | --- | --- | --- |
| Barry 2011 | Y | Social Cognitive Theory  Transtheoretical Model |  |  |
| Olson 2015 | Y | Social Cognitive Theory |  |  |
| Lubans 2009 | Y | Social Cognitive Theory |  |  |
| Barnason 2016 | Y | Social Cognitive Theory |  |  |
| Jennings 2014 | Y | Theory of Planned Behaviour |  |  |
| Volders 2019 | Y | Theory of Planned Behaviour  Precaution Adoption Process Model  Self-Regulation Model  I-Change Model |  |  |
| Lari 2018 | Y | Health Promotion Model | Y | Theory of Planned Behaviour |
| Ström 2013 | Y | Acceptance Commitment Theory  Transtheoretical Model |  |  |
| Verwey 2014  Van der Weegen 2015  Verwey 2016 | Y | 5 As model  Goal Setting Model |  |  |
| Harrison / Patel 2019 | Y | Prospect Theory |  |  |
| Ingram 2018 | Y | Self-Determination Theory | Y | Behaviour Change Technique (BCT) Taxonomy v1 |

| Hawkins 2019 | Y | Self-Determination Theory |  |  |
| --- | --- | --- | --- | --- |
| Givon 2016 |  |  | Y | Self-Determination Theory |
| Lorig 2010 |  |  | Y | Self-Efficacy Theory |
| Fife Schaw 2014 |  |  | Y | Social Cognition Model (doesn’t call it Social Cognitive Theory (SCT) nor reference to Bandura (1986) but likely to be SCT) |
| Bossen 2013 | Y | Operant behavioural principles (likely to be the same as Operant Learning Theory) |  |  |
| Kloek 2014 | Y | Goal Setting Theory |  |  |
| Brouwers 2017 |  |  | Y | Mentions behavioural change strategies including relapse prevention - but not referenced as behaviour change techniques / BCTs or Michie et al 2013 |
| Devi 2014 |  |  | Y | BCT taxonomy v1 |
| Sharma 2019 |  |  | Y | BCT taxonomy v1 |
| Alonso-Dominguez 2017/ 2019 |  |  | Y | Transtheoretical Model |
| Jones 2016 | Y | Social Cognitive Theory  Transtheoretical Model  Health Beliefs Model  Theory of Planned Behaviour |  |  |
